# Combined Mitral and Aortic Valvar Bioprosthesis Transcatheter Transapical Implant: First Description in Brazil

**DOI:** 10.5935/abc.20170118

**Published:** 2017-11

**Authors:** Roney Orismar Sampaio, Milena Ribeiro Paixão, Thais Taveira Miranda, Elinthon Tavares Veronese, José Honório de Almeida Palma, Flávio Tarasoutchi

**Affiliations:** Instituto do Coração (InCor) - Faculdade de Medicina da Universidade de São Paulo, São Paulo, SP - Brazil

**Keywords:** Transcatheter Aortic Valve Replacement, Heart Valve Prosthesis Implantation, Heart Valve Prosthesis

## Introduction

From the earliest days of the heart surgery, the valves, when the reparation was not possible, were replaced by prosthetics using cardiopulmonary bypass. The good results of these procedures are well known. In the recent years, minimally invasive alternatives have been developed, aiming to make possible the treatment of individuals under high risk of complications and death caused by the conventional procedure. In 2002, the first transcatheter valvar implantation was made, which revolutionized the treatment of severe aortic stenosis. The equipment, techniques and skills have progressively evolved since then. More recently, from 2009, transcatheter mitral valvar implants for treatment of the prosthesis dysfunction (valve-in-valve) started to be performed. Currently, the transcatheter valvar implantation is one of the fields of greater development in cardiology.

## Case Report

A male patient aged 72 was admitted to the emergency room with congestive heart failure of progressive worsening. He presented a history of rheumatic fever, coronary artery disease, atrial fibrillation, chronic renal failure (creatinine clearance of 58 mL/min/1,72 m^2^) and amaurosis secondary to the macular degeneration of the retina. He underwent two previous cardiac surgical procedures: mitral valvuloplasty and coronary artery bypass grafting in 1993 and mitral valve replacement by bioprosthesis and new myocardial revascularization in 1998. There was a great technical difficulty in the last procedure, due to multiple adhesions.

Upon physical examination at the entrance, he presented BP = 120/70 mmHg, HR = 60 bpm, irregular. Cardiac auscultation: hyperphonetic first heart sound, crescendo/decrescendo systolic murmur 5+/6+ in an aortic focus and holosystolic regurgitating murmur 3+/6+ in a mitral focus. The rest of the physical examination went without particularities.

An echocardiography was performed, which showed a left atrium of 74 mm, diastolic diameter of the left ventricle of 52 mm and systolic diameter of 32 mm, thickness of the septum and posterior wall of 15 mm, systolic pulmonary artery pressure of 53 mmHg and ejection fraction of 77%. The mitral bioprosthesis was calcified with average LA/LV gradient of 13 mmHg, area of 1.7 cm^2^ and important reflux. The aortic valve was calcified, with important stenosis (peak transvalvar gradient of 104 mmHg and average of 62 mmHg, valve area of 0.67 cm^2^) and discreet reflux.

The cineangiocoronariography identified saphenous vein graft to the occluded right coronary artery, mammary artery to the anterior pervious descending, and multiple lesions in the native beds of the right coronary and left anterior descending arteries.

**Figure 1:**
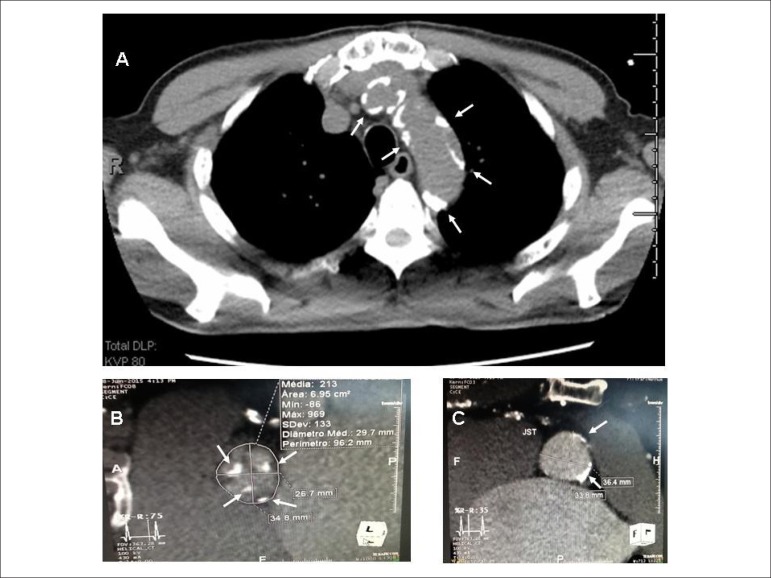
Computed thoracic tomography showing intense calcification of the ascending aorta (A), aortic valve (B) and sinotubular junction (C). Measurements of the valve diameter (A) used to define the size of the prosthesis and of the sinotubular junction (B).

The thoracic angiotomography showed valve calcium score of 3580 agatston and important coronary atheromatosis and ascending aorta (Figure 1).

There were no acute compensating factors for heart failure, except for congestion attributed to the aortic and mitral valvar disease. The surgical risk by EuroSCORE II was of 13.23%. Due to the high surgical risk and technical difficulties reported in the last surgery, a discussion was held by the "Heart Team", and they opted for the valvar double percutaneous treatment through transapical transcatheter implant and approach of the coronary lesions retrospectively, prioritizing the resolution of the hemodynamic condition of the patient.

**Figure 2:**
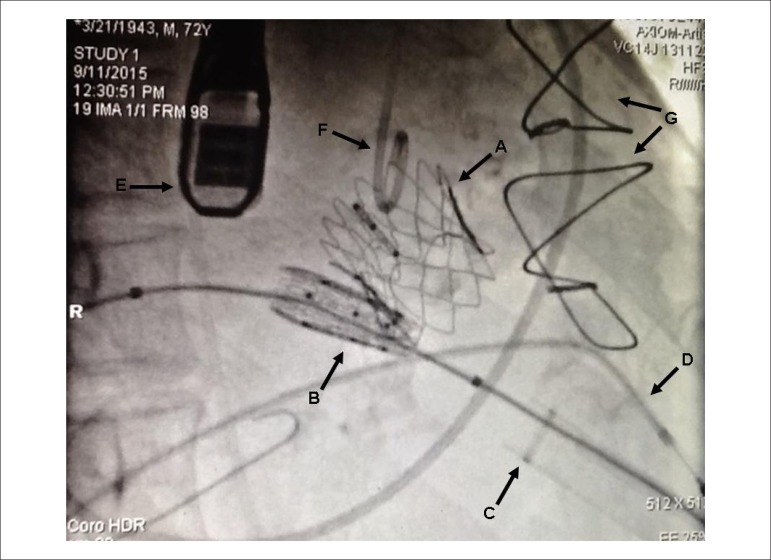
Radioscopy image showing Inovare-Braile bioprosthesis in aortic position no. 28 already implanted (A) and in mitral position no. 30, immediately after the implant (B). The sheath with guidewire for implantation of the mitral prosthesis (C), transvenous pacemaker electrode (D), transducer of the transesophageal echocardiography (E), pigtail catheter in the ascending aorta (F) and prior sternotomy wires (G) are observed.

On September 2015, the implantation of the aortic bioprosthesis and, after, of the mitral bioprosthesis were performed, both Inovare-Braile, numbers 28 and 30, respectively (Figure 2). The procedure occurred without complications. On the third post-operatory day, piperacillin and tazobactam were started, for the treatment of nosocomial pneumonia. He presented plateletopenia (up to 30,000/mm^3^), worsening of the acute renal insufficiency and moderate pericardial effusion, quickly reversed with the use of corticosteroids and usual clinical measures. Following clinical stabilization, the patient was discharged. The three-dimensional echocardiography after the discharge presented well-placed prosthesis, without significant periprosthetic reflux in the valve in the aortic position, peak LA/LV gradient 24 mmHg and average of 14 mmHg. On the mitral position, a moderate periprosthetic reflux was observed, average LA/LV gradient of 9mmHg, mitral area 1.5 cm^2^, SPAP 46 mmHg. There was clinical improvement for functional class II.

## Discussion

The dysfunction of the valvar bioprosthesis may be secondary to the degeneration of the leaflets due to wear, calcification or rupture, as well as the formation of *pannus* (host tissue), thrombus or perivalvular leak. The durability is smaller when in the mitral position, in young individuals, in the presence of prosthetic mismatch, renal failure and hyperparathyroidism.^1,2^

The increase in the use of biological prosthesis, associated to the increase of survival of operated individuals, have made surgical rapprochement increasingly common. The valve replacement surgery is, to this moment, the procedure of choice in cases of graft dysfunction, and it is associated to higher morbidity and mortality in relation to the first intervention. The technical difficulties of the surgical rapprochement may imply in longer period of cardiopulmonary bypass, need for transfusional support, and diaphragmatic paralysis by phrenic nerve injury, prolonged vasoplegia, aorto-coronary graft injury and increased risk of death.^1,2^

The predictors of higher risk of complication are: advanced age, cognitive dysfunction, peripheral vascular disease, chronic lung disease, renal failure, functional class IV heart failure by the New York Heart Association (NYHA), ventricular dysfunction, combined procedures, number of rapprochement, mitral valve replacement, emergency surgery, shock in the preoperative period, stent thrombosis, the presence of endocarditis and paravalvular abscess.^1,2^

The most used cardiac surgical risk scores in the daily practice are the STS and the EuroSCORE II. The former does not contemplate the risk calculation for double valvar replacement, as in the case at hand. The risk estimated by the EuroSCORE II was of 13.23%, indicating high risk of death.

One of the alternatives to the conventional aortic valvar surgery for individuals of high surgical risk is the transcatheter implant, known as Transcatheter Aortic Valve Implantation (TAVI), which is being performed since 2002.^3^ Since then, over 50,000 devices have been implanted throughout the world, with clinical outcomes that are similar to the surgery for patients with high or prohibitive surgical risk.^1^ The bioprosthesis may be expandable by balloon or self-expanding, both dependent on the presence of aortic valve calcification to prevent its displacement, however, with higher risk of perivalvar regurgitation in cases of extreme calcification.

The two most common pathways for the TAVI are transfemoral and transapical - a technique started in 2006, with access through minithoracotomy without the need for extracorporeal circulation. Although the transapical technique is more invasive, the advantages over the femoral pathway are: greater ease of valve implantation due to the proximity of the valve annulus of the cardiac apex, less manipulation of the aorta and peripheral arterial system reducing vascular complications and stroke.^1,4^ There is no impediment to the use of this technique in patients with previous myocardial revascularization.

The progress in the techniques and materials for transcatheter valve implantation in native valves allowed the strategy to be adapted for the treatment of dysfunction of biological prostheses, a technique called valve-in-valve. The aortic valve-in-valve procedures were the first to be performed, expanding the use of equipment and skills idealized for the TAVI. Since then, procedures with balloon-expansible and self-expansible prosthesis have been performed. Soon after, the method was expanded for mitral, pulmonary and tricuspid interventions.^1,5,6^

The first mitral valve-in-valve procedures were performed in 2009, initially, with balloon-expansible prosthesis and, after, also with self-expansible prosthesis. From 2011, implants over the post annuloplasty valve annulus began to be performed, known as valve-in-ring. Most recently, from 2014, the mitral transcatheter interventions on native valves began.^1,7^

Lastly, combined transcatheter procedures have been reported in the last few years.^8-10^ It is a treatment that requires further investigation, exclusively proposed in cases where the conventional surgical procedure is prohibitive. We suggest a multidisciplinary discussion with a "Heart Team" for each patient, aiming to design the best type of intervention individually and cautiously.

## Conclusion

There are few reports of combined intervention in disorders of aortic and mitral valves.^8-10^ This case was the first performed in Brazil with the implantation of the Inovare Braile national prosthesis, showing the huge potential for future interventions in selected patients.
